# Supplementary material for: The glycine N-methyltransferase amino-terminus regulates folate-dependent feedback inhibition and S-adenosylmethionine homeostasis
Source: J Biol Chem. 2026 Aug 19;302(10):113468. doi: 10.1016/j.jbc.2026.113468 (PMC13625792; doi:10.1016/j.jbc.2026.113468)
Supplement: Supplementary file 1 [file mmc7.docx]

Supplementary Figure S1. GNMT S9 phosphorylation (S9ph) varies across dietary and disease contexts. (A) GNMT S9ph occupancy estimates in mouse liver samples from ad libitum-fed (AL) and sulfur amino acid-restricted (SA) mice. The left panel shows raw peptide-pair occupancy estimates, and the right panel shows DEP-style VSN-normalized apparent occupancy estimates. Individual points represent biological samples, black diamonds indicate group means, and error bars indicate standard deviation. (B) Relative GNMT S9ph abundance in matched normal and tumor samples from an HBV-related hepatocellular carcinoma proteogenomic dataset. Violin plots show the distribution of relative phosphosite abundance across normal and tumor groups, with overlaid boxplots and individual sample points; the Wilcoxon rank-sum test P value is shown above the plot.

Supplementary Figure S2. Structural insights into the distal N-terminal tail and oligomeric state of human GNMT. (A) Overlaid structures of previously published human GNMT structure (PDB: 1R74, purple) and this study’s structure (PDB: 12YI, yellow), highlighting improved resolution of the distal tail region including the N-acetylvaline. (B) Representative electron density map supporting placement of the distal N-terminal tail of human GNMT. (C) Analytical ultracentrifugation analysis of GNMT in the presence (0.5 mM or 1 mM) or absence of SAM.

Supplementary Figure S3. Residue contacts with 5mTHF in the GNMT-binding pocket. (A) Ligand interaction diagram showing atomic contacts between 5mTHF-(Glu)_5_ and GNMT residues during the selected molecular dynamics trajectory. Interactions shown occurred for more than 30.0% of the simulation time over the selected trajectory from 0 to 1000 ns. Interacting residues and waters are labeled by chain and residue number, and dashed interaction lines indicate the interaction type and contact frequency. Interaction frequencies may exceed 100% when a single residue forms multiple simultaneous interactions with the same ligand atom. For example, arginine side chains can have multiple hydrogen-bond donors that contact a single hydrogen-bond acceptor. Residue classes and interaction types are indicated in the legend, including charged, hydrophobic, polar, water-mediated, pi-cation, and solvent-exposure interactions. (B) Number of interactions gained and lost between 5mTHF and residues of GNMT in the presence and absence of S9ph.

Supplementary Figure S4. Leucovorin dose response, methionine uptake, and metabolite measurements in GNMT-expressing cells. (A) Full leucovorin dose-response analysis of media sarcosine concentration in HepG2 cells expressing GFP control, full-length GNMT, GNMT Δ8, or GNMT S9E. Individual points indicate replicate measurements. (B) Western blot showing titration of pLEX304/07 AHCY lentivirus. The pLEX304 200uL condition was chosen for relevant experiments. (C) Cellular methionine uptake assay in HepG2 cells expressing GFP, full-length GNMT, or GNMT Δ8 after incubation with radiolabeled ^3^H-methionine. (D) Cellular methionine uptake assay in MIHA cells expressing GFP, full-length GNMT, or GNMT Δ8 after incubation with radiolabeled ^3^H-methionine.

Supplementary Figure S5. GNMT Δ8 expression alters global lysine methylation patterns. Immunoblot analysis of global lysine methylation marks in HepG2 and MIHA cells expressing GFP control, full-length GNMT, or GNMT Δ8. Blots show pan-monomethyl lysine (Kme1), dimethyl lysine (Kme2), and trimethyl lysine (Kme3) signal across expression conditions. β-tubulin serves as the loading control for figures 5A, 6B, and S5A, as these samples were derived from the same experiment and analyzed on the same blot.

Supplementary Figure S6. Transcriptomic remodeling induced by GNMT Δ8 expression in MIHA cells. (A) Principal component analysis of variance-stabilized RNA-seq counts from MIHA cells expressing GFP control, full-length GNMT, or GNMT Δ8. Each point represents an individual biological replicate, and shaded ellipses indicate the distribution of replicates within each expression condition. (B) Volcano plot of differential gene expression in MIHA cells comparing full-length GNMT with GFP control, with log2 fold change on the x-axis and −log10 adjusted *P* value on the y-axis. Upregulated, downregulated, and non-significant genes are indicated, with selected genes labeled. (C) Dot plot of pathway enrichment analysis from MIHA GNMT Δ8 versus full-length GNMT comparison, grouped by GO Biological Process, KEGG, and Reactome categories. Dot size indicates gene ratio, and color indicates −log10 FDR.
